# Small Pox in Pittsburgh and Its Vicinity

**Published:** 1829

**Authors:** 


26. *Small pox in Pittsburgh and its vicinity.*—Dr. Callaghan (Amer. Jour,) has published a short account of the small pox, which prevailed in the western parts of Pennsylvania in the winter of 1828–9. About 60 persons died of the disease in the city of Pittsburgh. The disease seems to have been mild and distinct—requiring the usual antiphlogistic measures carried to moderate degree.

A goodly number of persons reported to have been vaccinated took the disease, insomuch that the faith of the community in the preventive powers of vaccination, is considerably shaken. One young man came under my observation, who had been vaccinated ten or twelve years before by a most respectable practitioner in Philadelphia; he had a distinct and well-defined mark of a vaccine vesicle on the arm, yet he had the natural small-pox in the most regular manner, though mild.

Here there were a numerous family of young persons, none of whom caught the disease. They had all been formerly vaccinated; some six, some nine, some twelve, and some twenty years before. A boy who had not been vaccinated, caught the disease; during his convalescence, and throughout the greater part of his illness, a child, about three or four years of age, who had been formerly vaccinated, was much about him—she escaped the disease.

In a family who occupied a house, in the cellar of which some persons of colour resided, ill of the small-pox, three of the children were liable to the contagion; they were vaccinated and escaped.–302.

The disease was said to have been introduced by a negro from Canada, but Dr. L. insists that it depended on the state of the atmosphere. We must say, however, that in whatever mode this malady was at first produced, nothing seems to us better ascertained than that it is propagated by contagion.

